# Video-rate endocavity photoacoustic/harmonic ultrasound imaging with miniaturized light delivery

**DOI:** 10.1117/1.JBO.29.S1.S11528

**Published:** 2024-03-19

**Authors:** Donghyeon Oh, Hyunhee Kim, Minsik Sung, Chulhong Kim

**Affiliations:** Pohang University of Science and Technology, Medical Device Innovation Center, Departments of Electrical Engineering, Convergence IT Engineering, Mechanical Engineering, Medical Science and Engineering, Pohang, Republic of Korea

**Keywords:** photoacoustic, ultrasound, endocavity, transrectal, transvaginal

## Abstract

**Significance:**

Endocavity ultrasound (US) imaging is a frequently employed diagnostic technique in gynecology and urology for the assessment of male and female genital diseases that present challenges for conventional transabdominal imaging. The integration of photoacoustic (PA) imaging with clinical US imaging has displayed promising outcomes in clinical research. Nonetheless, its application has been constrained due to size limitations, restricting it to spatially confined locations such as vaginal or rectal canals.

**Aim:**

This study presents the development of a video-rate (20 Hz) endocavity PA/harmonic US imaging (EPAUSI) system.

**Approach:**

The approach incorporates a commercially available endocavity US probe with a miniaturized laser delivery unit, comprised of a single large-core fiber and a line beamshaping engineered diffuser. The system facilitates real-time image display and subsequent processing, including angular energy density correction and spectral unmixing, in offline mode.

**Results:**

The spatial resolutions of the concurrently acquired PA and harmonic US images were measured at 318  μm and 291  μm in the radial direction, respectively, and 1.22 deg and 1.50 deg in the angular direction, respectively. Furthermore, the system demonstrated its capability in multispectral PA imaging by successfully distinguishing two clinical dyes in a tissue-mimicking phantom. Its rapid temporal resolution enabled the capture of kinetic dye perfusion into an ex vivo porcine ovary through the depth of porcine uterine tissue. EPAUSI proved its clinical viability by detecting pulsating hemodynamics in the male rat’s prostate *in vivo* and accurately classifying human blood vessels into arteries and veins based on ${\mathrm{sO}}_{2}$ measurements.

**Conclusions:**

Our proposed EPAUSI system holds the potential to unveil previously overlooked indicators of vascular alterations in genital cancers or endometriosis, addressing pressing requirements in the fields of gynecology and urology.

## 1 Introduction

Genital cancer is a serious global health concern, comprising 14.8% of all reported global cancer cases in 2020.^1^ Specifically, prostate cancer stands as the most prevalent malignancy among the male population, with an estimated 1,414,259 cases in 2020, leading to 375,304 fatalities.^1^ Simultaneously, ovarian cancer ranks as the seventh most prevalent cancer and the fifth leading cause of death among women worldwide, accounting for 314,000 new cases and 207,000 deaths in 2020.^1^ Transrectal or transvaginal (together noted as “endocavity”) ultrasound (US) imaging is the most representative diagnostic imaging technique for visualizing deeply situated reproductive organs. By accessing these organs through the rectal or vaginal canal, endocavity US enables close examination, revealing clinical symptoms that may remain undetectable using standard transabdominal US imaging.^2^^,^^3^ This approach is often employed in the initial evaluation of malignant lesions in the ovaries or prostate, including tumor masses and cysts.^4^^,^^5^ Additionally, it aids in guiding precise needle placement during needle aspiration biopsy, a confirmatory step in cancer diagnosis.^6^^–^^8^ For its specialized application, the endocavity US probe is characterized by a long neck for insertion and features a microconvex transducer array at its distal end, providing a wide-angle fan-shaped view.

Although endocavity US is a valuable imaging tool for assessing genital abnormalities, it is not typically employed as the sole diagnostic test owing to its relatively modest diagnostic accuracy. Transrectal US exhibits a sensitivity of 53.3% and a specificity of 75% for prostate cancer,^9^ whereas transvaginal US demonstrates a sensitivity of 88% and a specificity of 79% for deep endometriosis.^10^ Thus, definitive diagnoses usually rely on a combination of diagnostic methods, encompassing physical examinations (vaginal/rectal palpation), blood tests, advanced medical imaging (e.g., computed tomography or magnetic resonance imaging), and when necessary, histological analysis following surgical removal.

Moreover, the integration of photoacoustic (PA) imaging with clinical US imaging is actively progressing in clinical studies.^11^^–^^20^ This integration enhances the ability to capture subtle pathological signs and improves diagnostic accuracy.^21^^–^^23^ As a hybrid optical and acoustical imaging modality, PA imaging provides unique photochemical contrast capabilities at previously uncharted depths within biological tissue,^24^^–^^29^ surpassing the capabilities of other optical imaging methods.^30^^–^^37^ It mirrors the US imaging process^38^^–^^42^ but utilizes pulsed laser excitation to induce the PA effect in tissue chromophores (e.g., hemoglobin, melanin, lipid, and collagen).^43^^–^^46^ The resultant thermal expansion generates an acoustic signal,^47^^–^^49^ which can be reconstructed into cross-sectional distributions of chromophores akin to US B-mode images.^50^^–^^52^ The technical similarities between PA and US imaging enable simultaneous acquisition of both types of images, with PA images offering valuable physiological information about the lesion’s photochemical characteristics, such as total hemoglobin and oxygen saturation.^52^^–^^57^ This ability to detect changes in oxygen saturation proves beneficial in diagnosing urological and gynecological conditions, identifying peritumoral hypoxia in genital cancer, and detecting menstrual cycle-related lesions such as endometrioma or uterine fibroids.

The main challenge in integrating PA imaging with endocavity US imaging is the size of the imaging probe, given the necessity of probe insertion into confined application sites. Most current PA imaging probes are primarily designed for on-skin use with direct cutaneous contact, overlooking size considerations. Of all components, the optical delivery module occupies the largest volume, necessitating miniaturization for endocavity use. Most PA imaging probes employ fiber bundles for high-energy laser pulse delivery, which are bulky and result in low optical coupling efficiency due to the dead spaces created when arranging thin fibers consecutively.^11^^,^^12^^,^^58^^,^^59^ This requirement for customized probe fabrication for embedding impacts the compatibility with clinical US imaging systems.^60^^–^^64^ Although some optical fiber-based endocavity PA imaging studies have been reported,^65^^–^^68^ they require customized fiber modifications or arrangements of multiple optical elements, which are less practical in clinical settings. Additionally, most previous probe designs adopt a bright-field illumination scheme, which necessitates a void space (referred to as “stand-off”) to position the optical beam in the US imaging plane. This configuration hinders the effective field of view (FOV) and weakens the acoustic signal’s strength. Direct laser exposure to the probe surface also results in notable photo-induced artifacts from the transducer elements, potentially obscuring PA contrasts originating from tissue chromophores.

In this study, we present an endocavity PA and harmonic US imaging (EPAUSI) system equipped with a miniaturized light delivery module composed of a single large-core optical fiber and line beamshaping engineered diffuser. By integrating this system with a commercially available endocavity US probe, we achieve a probe thickness of 25 mm. Our system, combined with a portable optical parametric oscillator (OPO) laser, provides real-time display of both PA and harmonic US at a video rate of 20 Hz, with a spatial resolution benchmarked at 318/291  μm in the radial direction and 1.22/1.50° in the angular direction. Multispectral PA imaging further enables spectral multiplexing of various chromophores as demonstrated through clinical dye separation *in vitro*. The system’s video-rate temporal resolution permits the observation of real-time dynamic changes in PA contrasts in clinically relevant scenarios such as clinical dye perfusion and pulsatile hemodynamic motions, as evidenced in experiments involving an *ex vivo* porcine ovary and an *in vivo* rat prostate. Finally, functional EPAUSI effectively measures oxygen saturation (${\mathrm{sO}}_{2}$) levels in the human arteries and veins of a healthy volunteer, highlighting its capability to detect pathological hypoxia in reproductive organs.

## 2 Materials and Methods

### 2.1 Endocavity Photoacoustic and Harmonic Ultrasound Imaging System Configuration

**Fig. 1:**
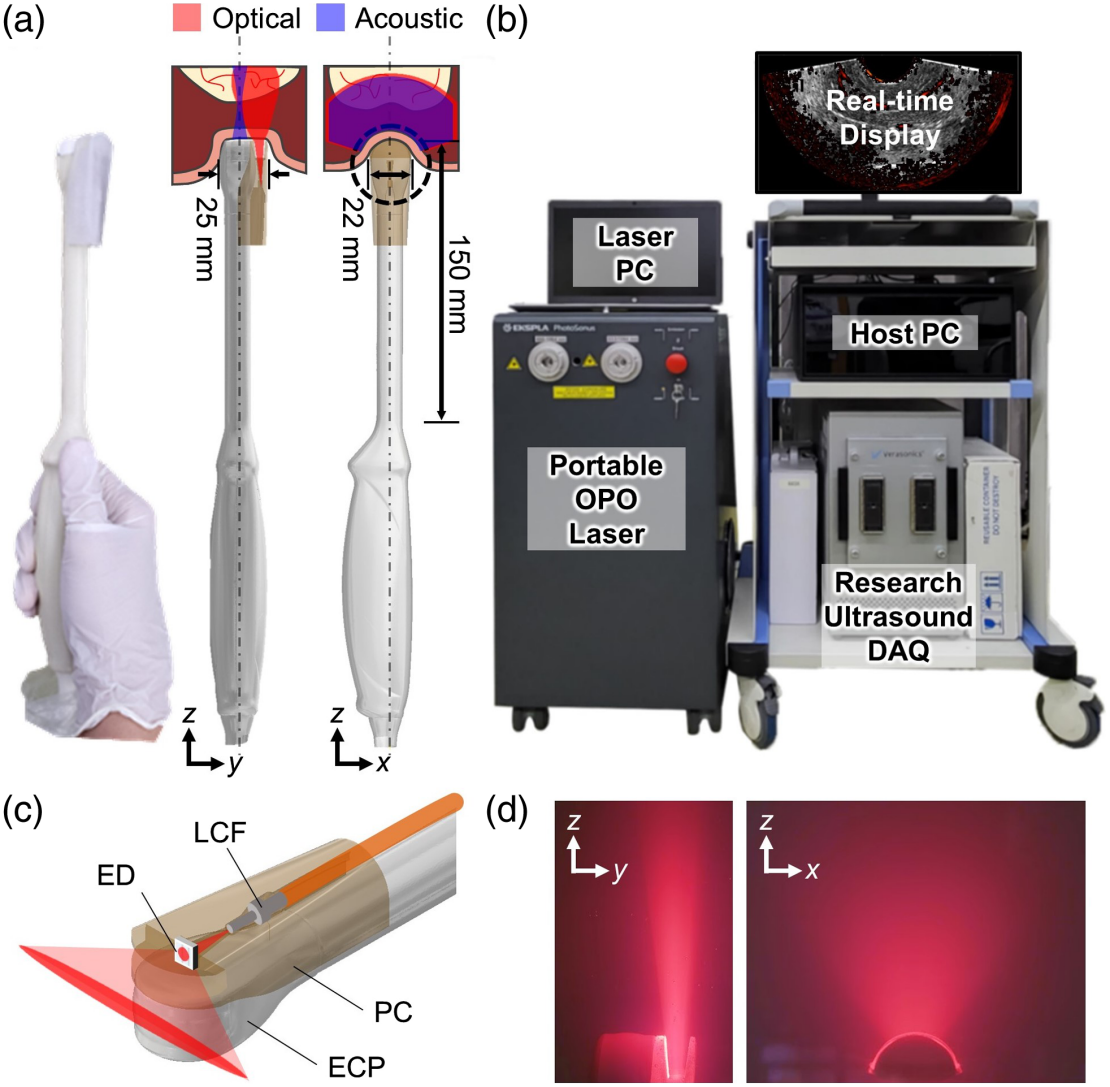
EPAUSI system configuration: (a) photograph and 3D rendering of EPAUSI probe assembled with optical casing. Optical and acoustic paths are depicted in red and blue, respectively. (b) System configuration of EPAUSI system on a cart. (c) Magnified rendering of EPAUSI probe head describing optical assemblies for line-beam shaping. (d) Photograph of a laser beam trajectory on the x-z and y-z planes imaged under a scattering suspension medium. PC, personal computer; OPO, optical parametric oscillator; DAQ, data acquisition unit; ED, engineered diffuser; LCF, large core optical fiber; ECP, endocavity ultrasound probe; and PC, probe casing.

Our EPAUSI system comprises a clinical endocavity probe for light delivery [as depicted in Fig. 1(a)], which is connected to a combination of a research US system (Vantage 256, Verasonics, Washington, United States) and a cart-mounted tunable pulse laser (PhotoSonus, Ekspla, Lithuania) [as shown in Fig. 1(b)]. For both PA and US acquisitions, we employed a commercially available endocavity US probe (6EIX, Humanscan, Republic of Korea) with a center frequency of 6 MHz and a 95% bandwidth. This probe features a microconvex transducer array positioned along the periphery of a 10 mm-radius circular edge, offering a wide-angle 160 deg fan-shaped FOV beneath the application site. In light of the practical scenario involving direct contact with a canal wall, we implemented a dark-illumination scheme, ensuring that the transducer surface is not directly exposed to a laser beam reflected from the tissue surface. This dark-field illumination scheme presents several practical advantages. First, it eliminates the need for a transparent standoff layer filled with water or gel to clear the light path, resulting in the efficient reduction of the overall probe size by economizing the void volume. Moreover, the removal of the standoff layer helps maintain the signal intensity by reducing subsidiary attenuation and preserving the original FOV. Finally, this scheme prevents the direct exposure of the laser beam to the probe elements, mitigating strong photoinduced artifacts generated from the probe lens surface, which often interfere with PA contrast from microvessels at shallow depths. To further minimize PA artifacts arising from the transducer surface, we replaced the probe’s acoustic lens with a white one. To preserve the identical physical properties and geometrical form factors of the transducer, the replaced lens was crafted using the same elastomer and lens mold as employed in the original lens fabrication process, with the only variation being in color through adjustments in the pigment ratio.

For laser transport, we employed a large-core optical fiber, significantly reducing the size of the laser delivery module while achieving superior optical coupling efficiency (approximately 90%), in contrast to using a fiber bundle (approximately 50%). Specifically, a 1 mm core size multimodal fiber (Optibase, South Korea), equipped with air-gapped SMA-905 fiber connectors and bare FC/PC ferrules on each end, was coupled to a customized fiber coupling system within the portable OPO laser system (operating at a wavelength of 660 to 1064 nm, a pulse width of 3 to 5 ns, and a pulse repetition rate of 20 Hz). This fiber delivered the laser beam to the distal end of the endocavity probe, and the beam was directed into a line beam-shaping engineered diffuser (EDL-150, VIAVI Solutions, Arizona, United States) installed in front of the fiber outlet [as depicted in Fig. 1(c)]. The initially circular-shaped beam diverged into a single direction as it passed through the engineered diffuser, eventually transforming its pattern into a line shape [Fig. 1(d)]. These optical components were integrated within a biodegradable 3D printed casing, which was attached to the side of the probe head. To maximize the PA signal, the fiber channel within the casing was slightly tilted toward the imaging plane. Consequently, the total thickness of the probe head was 25 mm.

### 2.2 Real-time, Video-rate PA and Harmonic US Image Processing

**Fig. 2:**
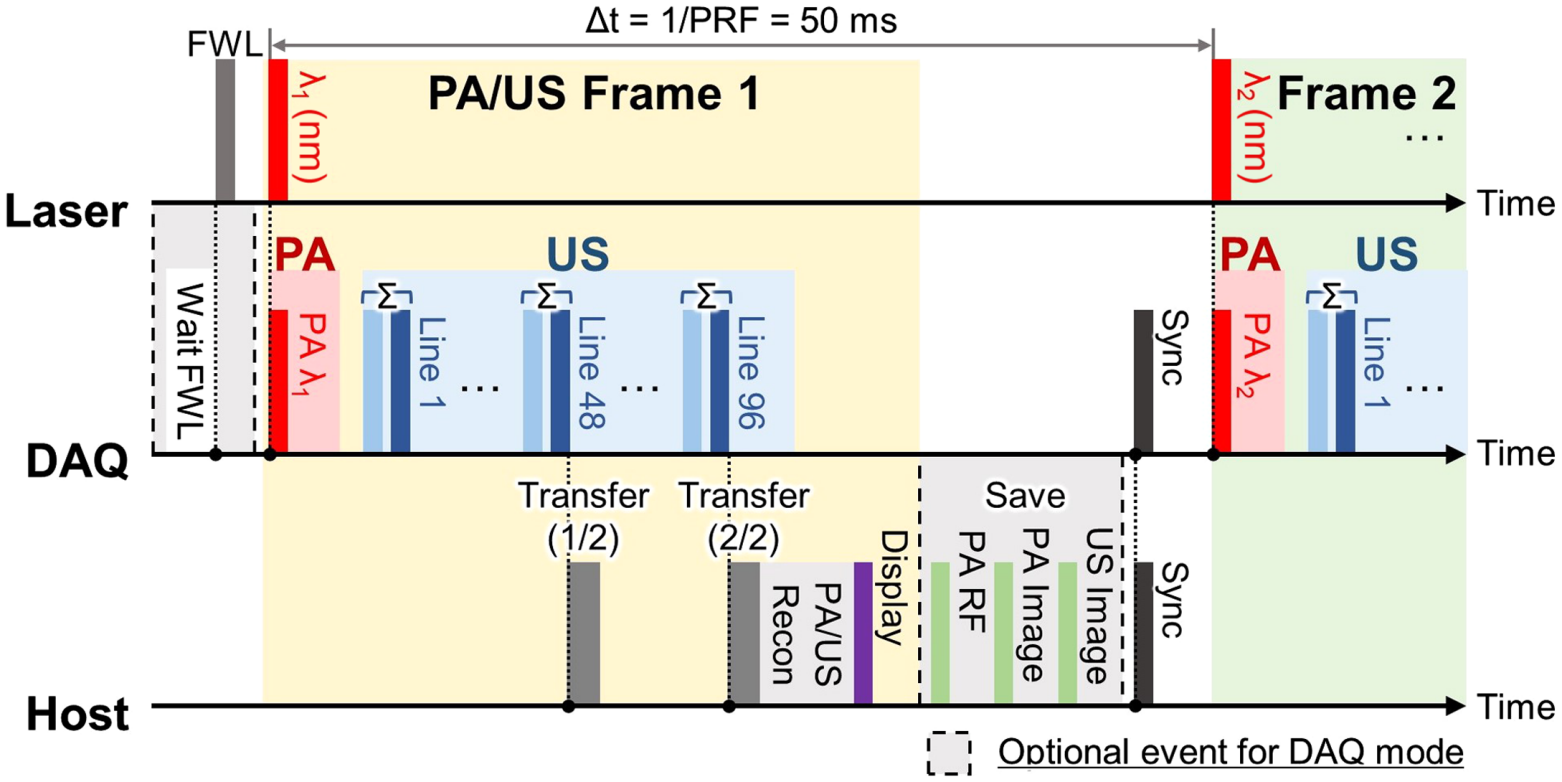
The 20 Hz video-rate timing sequence of multispectral PA and harmonic US imaging. FWL, first wavelength laser trigger; PRF, pulse repetition frequency; DAQ, data acquisition unit; and RF, radiofrequency data.

The signal acquisition, image reconstruction, and real-time display followed the event sequence timing outlined in Fig. 2. The acquisition of PA signals commenced with a laser trigger synchronized to a laser shot. This was swiftly followed by the acquisition of US signals, in which we implemented a pulse inversion harmonic US imaging technique to enhance the resolution and tissue contrast (Fig. S1 in the Supplementary Material). This method involves transmitting a pair of normal and inverted US pulses at each location, subsequently isolating the harmonic signal by canceling out the fundamental frequency signal. To match the probe’s bandwidth, we designed a pair of normal and inverted bipolar 4 MHz US transmit pulses, which ultimately resulted in an 8 MHz harmonic signal after the successful cancellation of the fundamental frequency signal by adding the radiofrequency (RF) signal pairs.

For each US frame, 96 line-focused US beams swept across the entire FOV, with the transmit pairs being sequentially positioned to minimize tissue motion between the two acquisitions. In total, 1 PA receive event and 192 harmonic US receive events (comprising 96 pairs) were accommodated within the elapsed time between two laser emissions, amounting to 50 ms. Unlike US signal reception involving active pulse transmission, PA signal reception experiences greater attenuation in both optical and acoustical domains. Accordingly, an additional gain was allocated for the PA acquisition event, utilizing a programmable variable-gain preamplifier consisting of a time-gain compensator (TGC), programmable gain amplifier (PGA), and low noise amplifier (LNA) relay. Although the TGC was adjustable, it was maintained at the maximum value across all depths to ensure consistency and maximize the preservation of deep PA signals. Additionally, an extra 12 dB gain (PGA: 30 dB; LNA: 24 dB) was selectively applied in PA acquisition compared with harmonic US acquisition (PGA: 24 dB; LNA: 18 dB), with the preamplifier settings remaining constant throughout the experiments. The acquired PA and US RF data were transferred twice during a single frame acquisition to the host PC for online image reconstruction, utilizing a real-time Vantage reconstruction algorithm (Verasonics, WA, USA). Consequently, a pair of PA and harmonic US images was displayed on the screen at a real-time frame rate of 20 Hz. For data export and subsequent offline processing, such as energy density compensation and spectral unmixing (as detailed in Sec. 2.3), a conditional loop was activated. This loop involved an additional trigger event and a data save event. Operating in a multiwavelength mode, the laser system emitted an extra trigger of 8 microseconds in advance of the emission of the first wavelength of the laser pulse, allowing the US system to organize the data in the order of the laser pulse relay. After repeating the acquisition event as commanded by the user, the multi-frame PA RF, PA image, and harmonic US image data were saved, and the system returned to the real-time mode.

### 2.3 Offline Angular Energy Density Correction and Spectral Unmixing

**Fig. 3:**
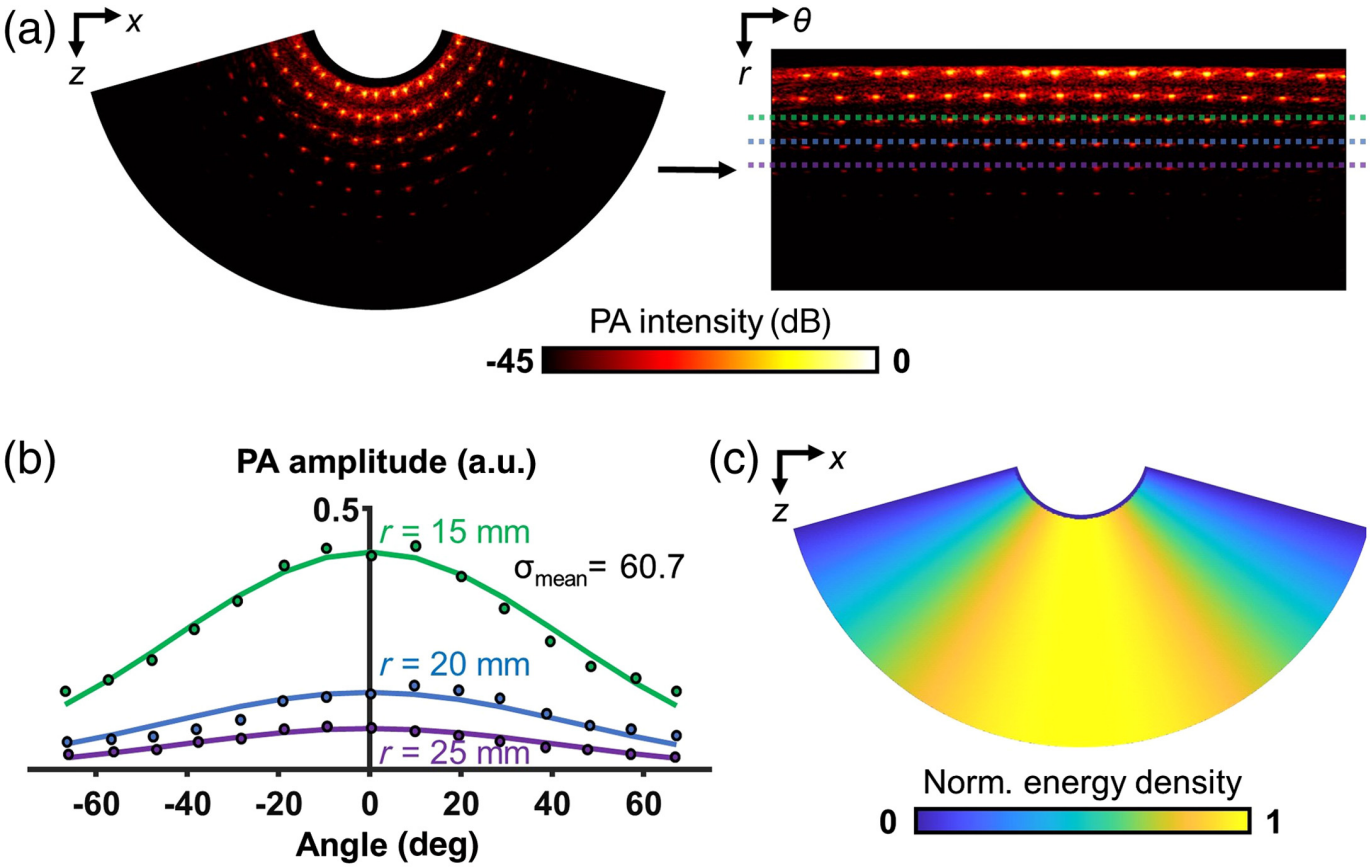
Estimation of angular energy density distribution using a tissue-mimicking phantom: (a) PA imaging result of ${\mathrm{TiO}}_{2}$-gelatin phantom (45 dB). To build a fluence distribution map based on angles, the image was changed from Cartesian coordinates to polar coordinates. (b) PA amplitude distribution by angles. (c) Estimated angular fluence distribution map.

We observed an angular deviation in laser intensity between the center and the sides of the wide-angle FOV. Consequently, we conducted post-processing on the PA images to achieve uniform PA intensity along the angular direction. Utilizing the original PA image of a tissue-mimicking phantom (as described in Sec. 3.1), we estimated the angular energy density distribution by analyzing the PA intensity of radial-patterned string targets. Initially, the PA image was transformed from Cartesian coordinates to polar coordinates. Subsequently, point targets spanning between ±70  deg and 15 to 25 mm in depth, along with their neighboring pixels, were windowed [as illustrated in Fig. 3(a)]. We calculated to the average amplitudes of the top 10% of pixels from each window, sorted them by depth, and generated an angular distribution of PA amplitude, as shown in Fig. 3(b). We fitted Gaussian curves to the measured data points, resulting in a consistent standard deviation of approximately 60 at all depths. Based on this observation, we created an estimated angular energy density map that covered the entire FOV. This map was generated by transforming the rectangular Gaussian distribution block into Cartesian coordinates, as depicted in Fig. 3(c). Subsequently, all experimental PA images were homogenized using inverse compensatory weighting based on the estimated angular energy distribution map.

We conducted linear spectral unmixing on the multispectral PA images to distinguish between multiple contrasts originating from distinct chromophores, such as clinical dyes and oxy and deoxyhemoglobin (as detailed in Secs. 3.2 and 3.5). Multispectral PA amplitudes at a specific location represent a combination of PA contrasts from various chromophores with distinct spectral properties, which are expressed using the following equation:

$$P=[\begin{array}{c} p(\lambda_{1})\\ p(\lambda_{2})\\ \;\vdots \\ p(\lambda_{n}) \end{array}]=[\begin{array}{cccc} \mu_{1}(\lambda_{1}) & \mu_{2}(\lambda_{1}) & \ldots & \mu_{m}(\lambda_{1})\\ \mu_{1}(\lambda_{2}) & \mu_{2}(\lambda_{2}) & \ldots & \mu_{m}(\lambda_{2})\\ \vdots & \vdots & \ddots & \vdots \\ \mu_{1}(\lambda_{n}) & \mu_{2}(\lambda_{n}) & \ldots & \mu_{m}(\lambda_{n}) \end{array}][\begin{array}{c} c_{1}\\ c_{2}\\ \vdots \\ c_{m} \end{array}]=MC,$$ (1)

where P represents a single-column PA amplitude vector composed of $p(\lambda_{i})$; M is a [n×m] matrix representing the molecular extinction coefficients and is composed of $\mu_{j}(\lambda_{i})$; and C is a single-column vector representing relative concentrations and is composed of $c_{j}$, where i and j denote the arbitrary indices of wavelengths and chromophores, both ranging from 1 to n and 1 to m, respectively. Referring to their measured molecular optical extinction coefficient available online (Ref. 69; Oregon Medical Laser Center, Oregon, United States), we inversely calculated the local concentration of the chromophores by implementing a pseudo-inverse matrix scheme:^18^

$$C=(M^{+})P={\left(M^{T}M\right)}^{-1}M^{T}P.$$ (2)

Considering the clinical scenario in which the probe is manually maneuvered by hand, we reduced the number of wavelengths (n) to maximize the spatial correlation among the PA images. To enhance the accuracy of ${\mathrm{sO}}_{2}$ measurements, we estimated an optical fluence for each original spectral PA image, and we performed preliminary compensation prior to its utilization as input for spectral unmixing. We divided the provided PA image into 1 cm depth increments, and within each step, we determined the mean value of the upper 50% of PA signals. These values were fitted with an exponential curve, assuming that optical fluence attenuation follows Beer–Lambert’s law. Distinct attenuation coefficients were obtained for each wavelength, and the respective PA images were inversely compensated by their corresponding exponential curves. Data post-processing was conducted using a custom script developed with MATLAB (MATLAB R2021a, MathWorks, Massachusetts, United States).

## 3 Results and Discussion

### 3.1 Spatial Resolution Benchmarking in ${\mathrm{TiO}}_{2}$-gelatin Phantom

**Fig. 4:**
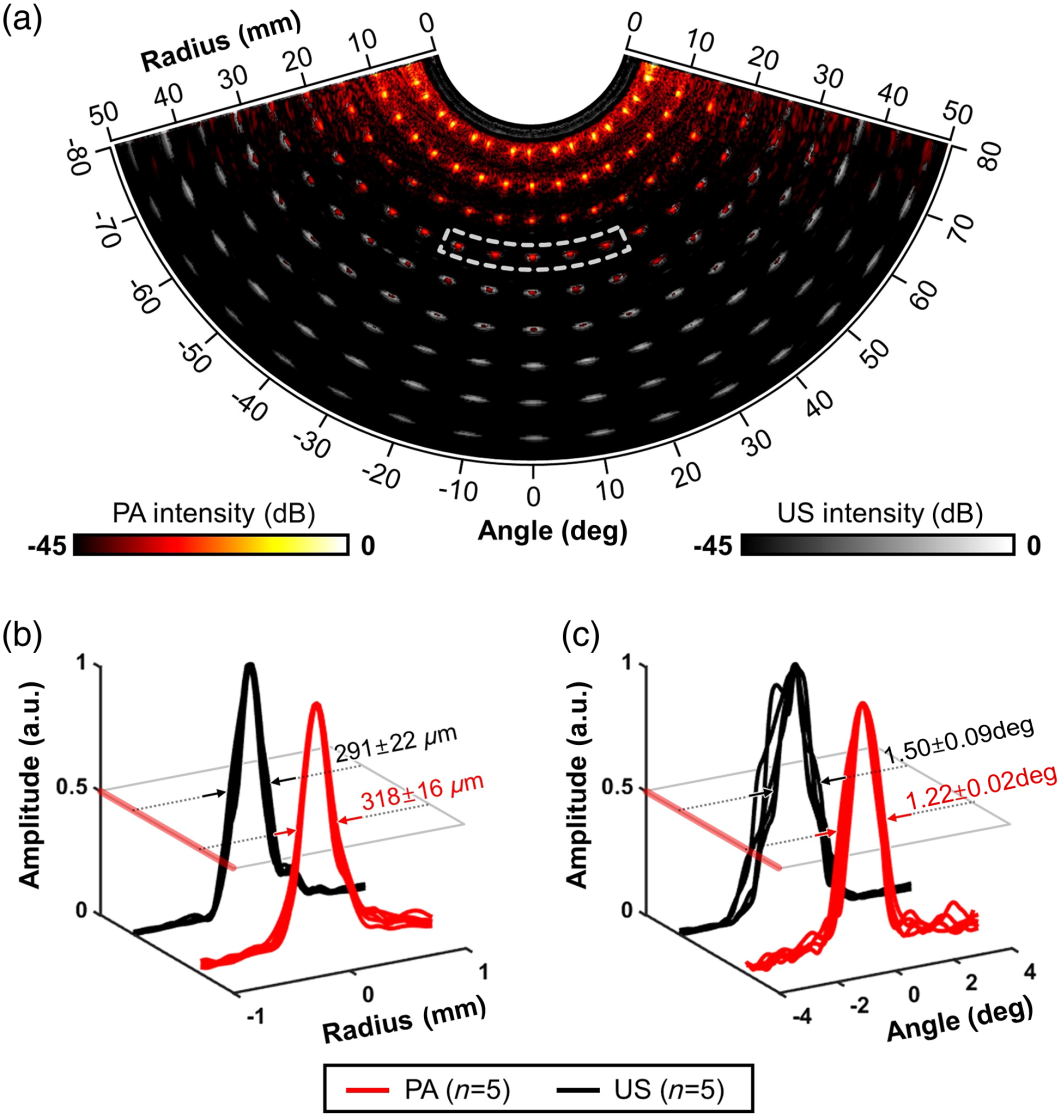
EPAUSI spatial resolution measured from a tissue-mimicking phantom. (a) overlaid PA and harmonic US images highlight the spatially correlated contrasts from points in a radial pattern. Quantification of (b) radial and (c) angular resolution from line profiles of five 20-mm depth point targets, marked with dashed white box in panel (a). Mean ± standard error.

The measurement of the image resolution and angular energy density distribution was conducted using an *in vitro* experiment with a tissue-mimicking phantom. To create the phantom mold, transparent acrylic boards were designed, and holes were evenly punched on both sides of the side face in a radial pattern, spaced at 10 deg intervals in the angular direction and 5 mm intervals in the depth direction. A 100  μm black nylon string was threaded through these holes to serve as the imaging target. For the medium, a gelatin solution was prepared by adding 16.7 w% gelatin to water, which was thoroughly dissolved through heating at 60°C. The medium’s optical turbidity was achieved by adding 1  g/L of ${\mathrm{TiO}}_{2}$ while constantly stirring.^70^ This prepared medium was subsequently poured into the threaded mold and allowed to cool to room temperature, solidifying into a gel phantom. To ensure complete contact with the cylindrical surface of the probe, a groove with a 12.5 mm radius was created by dipping a cylindrical mold during fixation. For imaging, the assembled endocavity probe was acoustically matched with the phantom using a droplet of water and securely fixed upright onto the groove, with adjustments guided by a real-time harmonic US view. PA images were acquired at 700 nm with an energy density of $20\;\mathrm{mJ}/cm^{2}$, adhering to the American National Standards Institute (ANSI) criteria for maximum permissible exposure (MPE) at NIR wavelengths. As a result of this experiment, the simultaneous acquisition of PA and harmonic US B-mode images with a strong spatial correlation of the point targets was achieved, as illustrated in Fig. 4(a) and Fig. S2 in the Supplementary Material. The PA contrast appeared to be evenly distributed across the full 160 deg fan-shaped FOV scan range, similar to the harmonic US images, which were reconstructed with geometric homogeneity. However, contrary to the US image contrast, which extended down to 50 mm, the PA contrast logarithmically decreased with depth and reached the noise-equivalent depth at approximately 35 mm due to optical attenuation in the phantom media.

In terms of spatial resolution, we observed that the line profile of the point targets broadened with increasing the depth. The distinctive shape of the array dimensions, being convex, led us to measure the radial and angular resolutions in polar coordinates, in contrast to the lateral and axial resolution measurements in Cartesian coordinates. We obtained line profiles of the five central point targets at a depth of 20 mm, considering the elevational focal length of the probe. The maximum amplitude projections in the radial and angular directions were normalized, and the peak widths were measured following the full-width–half-maximum criteria [as depicted in Figs. 4(b) and 4(c)]. The radial resolution, which was narrower in harmonic US (291±22  μm) compared with PA (318±16  μm), can be attributed to the inherent disparity in carrier frequency between PA and US signals. PA signals are modulated by the fundamental frequency of the transducer (6.25 MHz), whereas harmonic US signals are modulated at the second harmonic frequency of the transmit pulse (8 MHz). The angular resolution of PA (1.22±0.02 deg and 639±11  μm in lateral) was finer than that of harmonic US (1.50±0.09  deg and 785±49  μm in lateral), as shown in Fig. 4(c). This observation can be explained by the greater limited view effect induced by the transducer’s geometry. Unlike the round trip travel of US signals, PA signals travel in one direction, and PA images are affected by the limited acceptance angle of the piezo elements. Additionally, the curved arrangement of piezoelements in microconvex probes results in a sparser acceptance angle overlay, contributing to a more pronounced limited view effect. Consequently, PA images may provide a better resolution in depicting microvessels, which can be particularly useful in identifying cancerous angiogenesis.

### 3.2 Spectral Unmixing of Multiplexed Clinical Dyes *In Vitro*

**Fig. 5:**
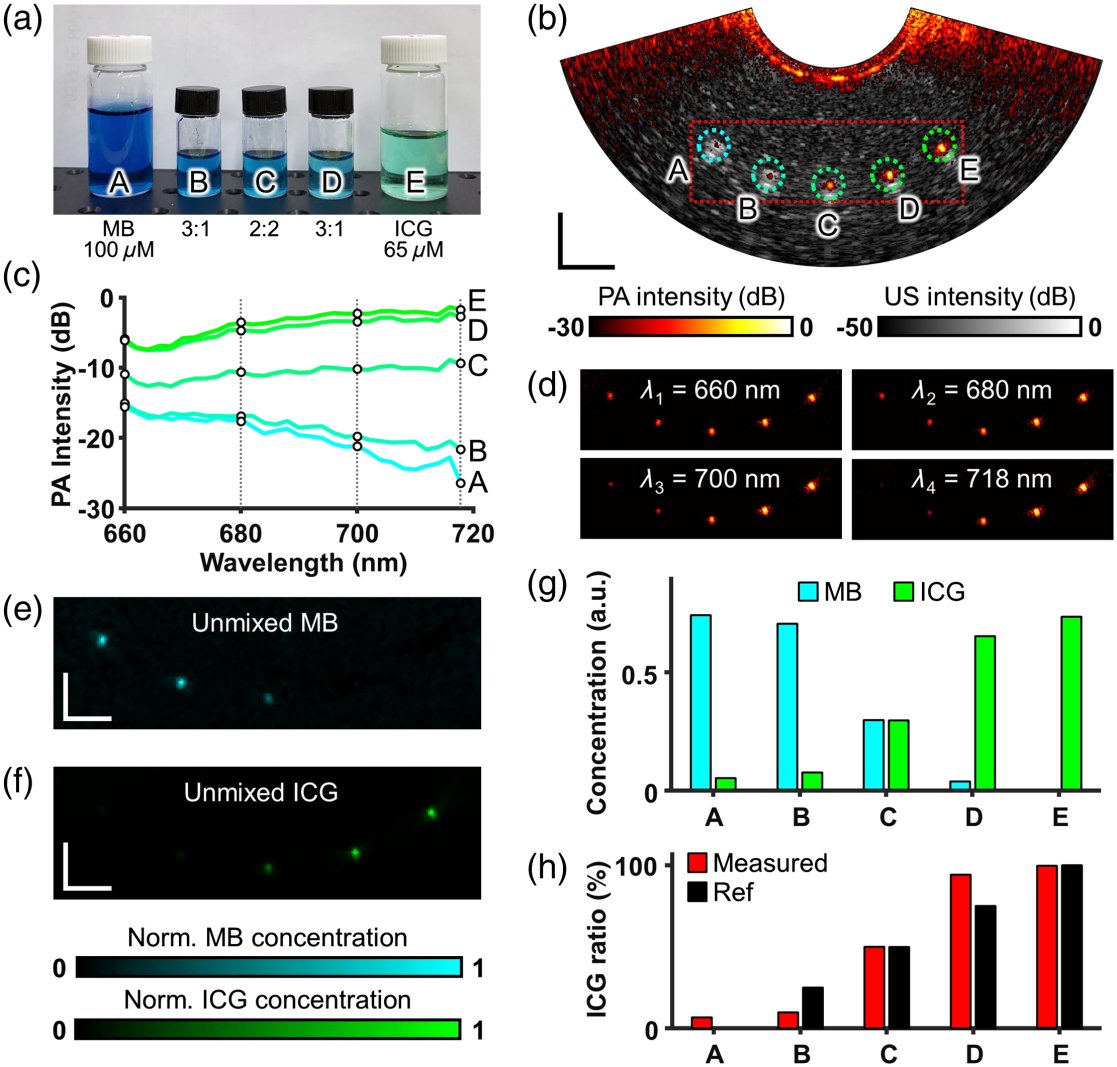
Differentiation of multiplexed clinical dye contrast with multispectral EPAUSI: (a) a photograph of five MB – ICG cocktail samples arranged in order of ICG constituent ratio. (b) An overlaid PA and US image of the microtubing phantom filled with five samples. Scale bar = 10 mm. (c) PA spectroscopy of five samples in the NIR range. (d) A series of PA images corresponding to four wavelengths (660, 680, 700, and 718 nm) involved in spectral unmixing. Multiplexed mapping of (e) a normalized MB concentration and (f) a normalized ICG concentration. Scale bar = 5 mm. (g) Quantified MB and ICG concentration from each corresponding tubing location. (h) Measured ICG constituent ratio and ground truth.

To validate the molecular imaging capabilities of our EPAUSI system, we conducted a study in which we analyzed the constituent ratio of two Food and Drug Administration (FDA)-approved contrast dyes using multispectral PA imaging under *in vitro* conditions. Multispectral PA imaging, which leverages the distinctive optical spectral characteristics of chromophores in biological tissues, is a valuable technique for distinguishing the molecular-specific distribution of substances. This technique is widely employed in the current clinical translation of PA imaging. We prepared aqueous solutions of 65  μM methylene blue (MB) and 100  μM indocyanine green (ICG) and adjusted their concentrations to achieve absorbance levels comparable to that of hemoglobin in the NIR range, as documented on the Oregon Medical Laser Center’s spectral data repository.^69^ To mimic the quantification of oxygen saturation, we mixed the MB and ICG solutions in volume ratios of 3:1, 2:2, and 1:3 [Fig. 5(a)]. Using a 27G syringe, these mixtures were subsequently injected into silicone microtubing threaded into the ${\mathrm{TiO}}_{2}$-gelatin phantom, as described in Sec. 2.1, with the MB content decreasing from left to right. To minimize variations in optical attenuation, all tubings were placed at the same depth (25 mm). For multispectral PA imaging, we selected 20 wavelengths at 2 nm intervals, ranging from 660 nm to 718 nm, as this range exhibited the most significant spectral absorbance variations for the two dyes.

In the resulting PA and harmonic US images obtained at each wavelength, the upper and lower boundaries of the tubing were clearly visible in the harmonic US images, whereas the PA contrast of the dye appeared as small dots in the center of the lumen in the PA images [see Fig. 5(b)]. As the wavelength changed from 660 to 720 nm, distinct spectral trends in the PA contrast were observed within the tubing samples containing MB and ICG. Specifically, MB exhibited a consistent diminishing pattern, whereas ICG displayed a consistent increase [see Fig. 5(c)]. For practicality, we performed spectral unmixing using only four out of the total 20 multispectral image ensembles. These four images were selected from the quartile range at wavelengths 660, 680, 700, and 718 nm [see Fig. 5(d)]. The resultant unmixed distribution images of MB and ICG demonstrated a consistent rise and decline, respectively, which strongly correlated with the actual fractional composition of each dye [see Figs. 5(e) and 5(f)]. We quantified the upper 10% pixel intensity within the designated region of interest (ROI) within each tube. This analysis allowed for clear discrimination between MB and ICG in the pure tubing samples, whereas an equal constituent ratio was observed in the 2:2 cocktail tubing [see Fig. 5(g)]. Furthermore, when we calculated the fractional ICG concentration from the unmixed MB and ICG images, a monotonic increase was highlighted; it closely correlating with the actual fractional ICG concentration [see Fig. 5(h)]. However, the values for B and D, which should exhibit a volumetric ratio of 25% and 75%, respectively, appeared to deviate from the most representative reference ratio among the measured values. This discrepancy may stem from variances between the reference molar absorption coefficient used in the pseudoinverse calculations and the actual absorption coefficients of ICG and MB.

### 3.3 ICG Infusion Kinetics into Ex Vivo Porcine Ovary

**Fig. 6:**
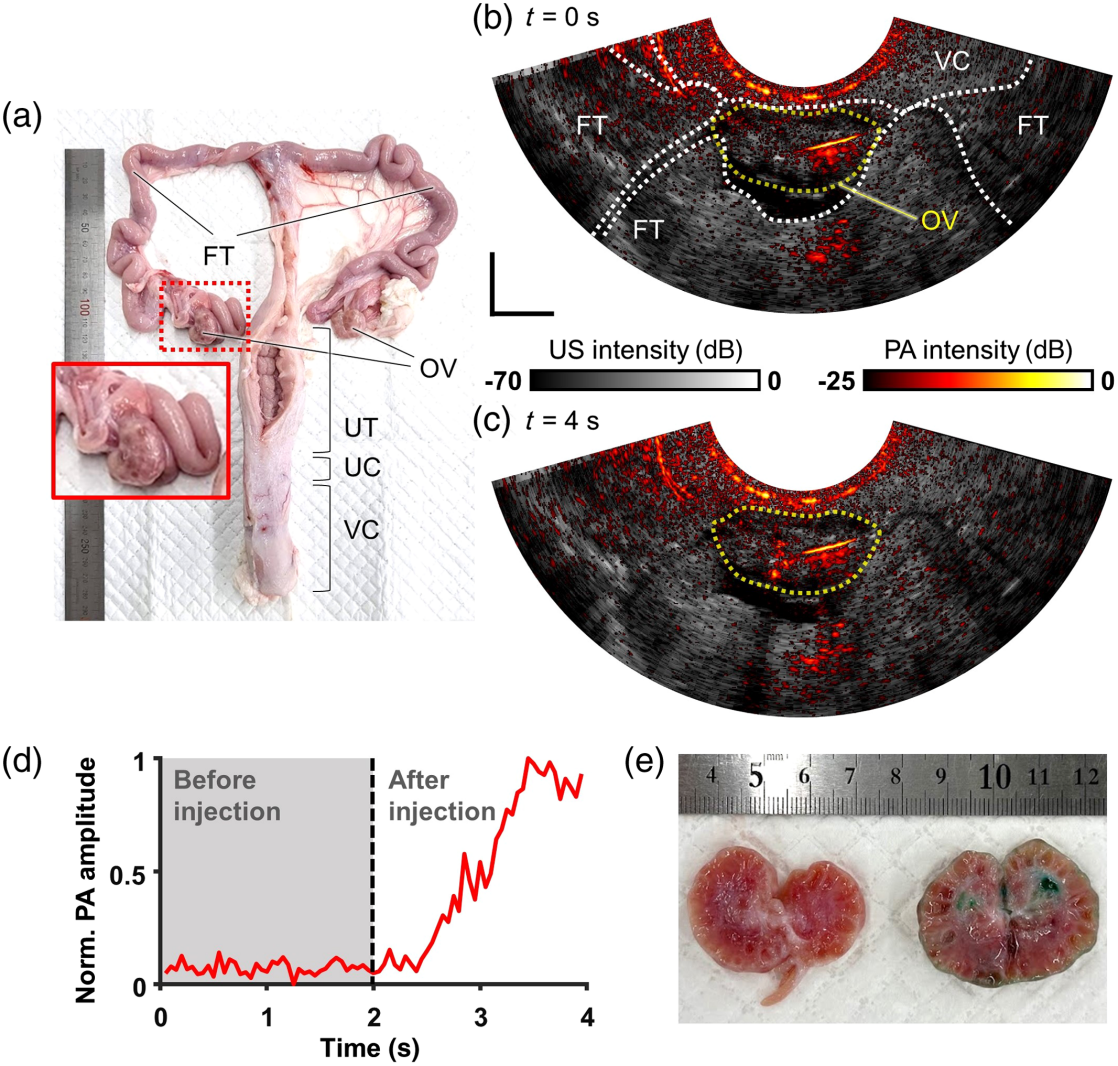
*Ex vivo* imaging of a porcine ovary with ICG infusion: (a) a photograph of a porcine female genital. A red inset image shows a magnified appearance of a porcine ovary in the end of a long FT. Comparative overlaid PA and harmonic US images of a tissue-surrounded ovary (b) in the preinjection state and (c) after ICG injection highlights the PA contrast of infused ICG (Video 1). (d) ICG infusion dynamics under the conditions of increasing PA amplitude inside the ovary. (e) A photograph of an ovarian hemisection after ICG infusion with topical ICG stain inside the ovary. UT, uterus; UC, uterus cervix; and VC, vaginal canal. Scale bar = 10 mm.

To assess the feasibility of using EPAUSI for transvaginal applications, we conducted real-time imaging of the dynamic ICG infusion into *ex vivo* female porcine genital tissue. In gynecological conditions necessitating surgical intervention, such as genital cancer or endometriosis, ICG is frequently employed for intraoperative fluorescent staining of malignant lesions, such as tumor masses or sentinel lymph nodes. For this experiment, we utilized a freshly excised porcine uterus, which included the ovary, fallopian tube (FT), uterus, uterine cervix, and vaginal canal [refer to Fig. 6(a)]. To replicate the clinical scenario, we enveloped the lower and surrounding regions of the excised ovary with FT tissue and subsequently covered them with an unfolded vaginal tissue flap. The EPAUSI probe was positioned over the vaginal tissue, and PAUS imaging was performed to penetrate through these tissue layers. Following probe positioning, harmonic US images clearly visualized the ovary at a depth of 23 mm, along with the adjacent FT tissues and the upper vaginal tissue layer. Subsequently, a 27G syringe needle was guided into the center of the ovary using real-time US imaging. Both 700 nm PA and harmonic US images were acquired at a rate of 20 Hz for a duration of 4 s, starting simultaneously with the injection of 3 mL of a 200  μM ICG solution through the needle.

Before the injection, the syringe needle piercing through the ovarian membrane and into the ovarian center was clearly visualized in both the 700 nm PA and US images [see Fig. 6(b)]. However, upon the initiation of ICG perfusion, the PA image exclusively displayed the formation of a strong PA contrast cloud at the distal tip of the needle. This PA contrast area continued to expand over a span of 2 s, whereas such an observation was not evident in the US image [see Fig. 6(c) and Video 1]. We extended our analysis to compare the temporal changes in PA and US amplitudes. This involved selecting an ROI along the ovarian boundary and calculating the upper 10% average of pixel amplitudes from both images. As a result, the PA signal exhibited a remarkable increase of up to 10-fold within 2 s from the point of injection, compared with the pre-injection levels [see Fig. 6(d)]. After acquiring the images, the ovary used in the experiment was dissected into halves. The localized ICG stain was distinctly observable in a location corresponding to where the ICG cloud was evident in the PA image [see Fig. 6(e)].

### 3.4 *In Vivo* Monitoring of Rat Prostate Vessel Pulsation with EPAUSI

**Fig. 7:**
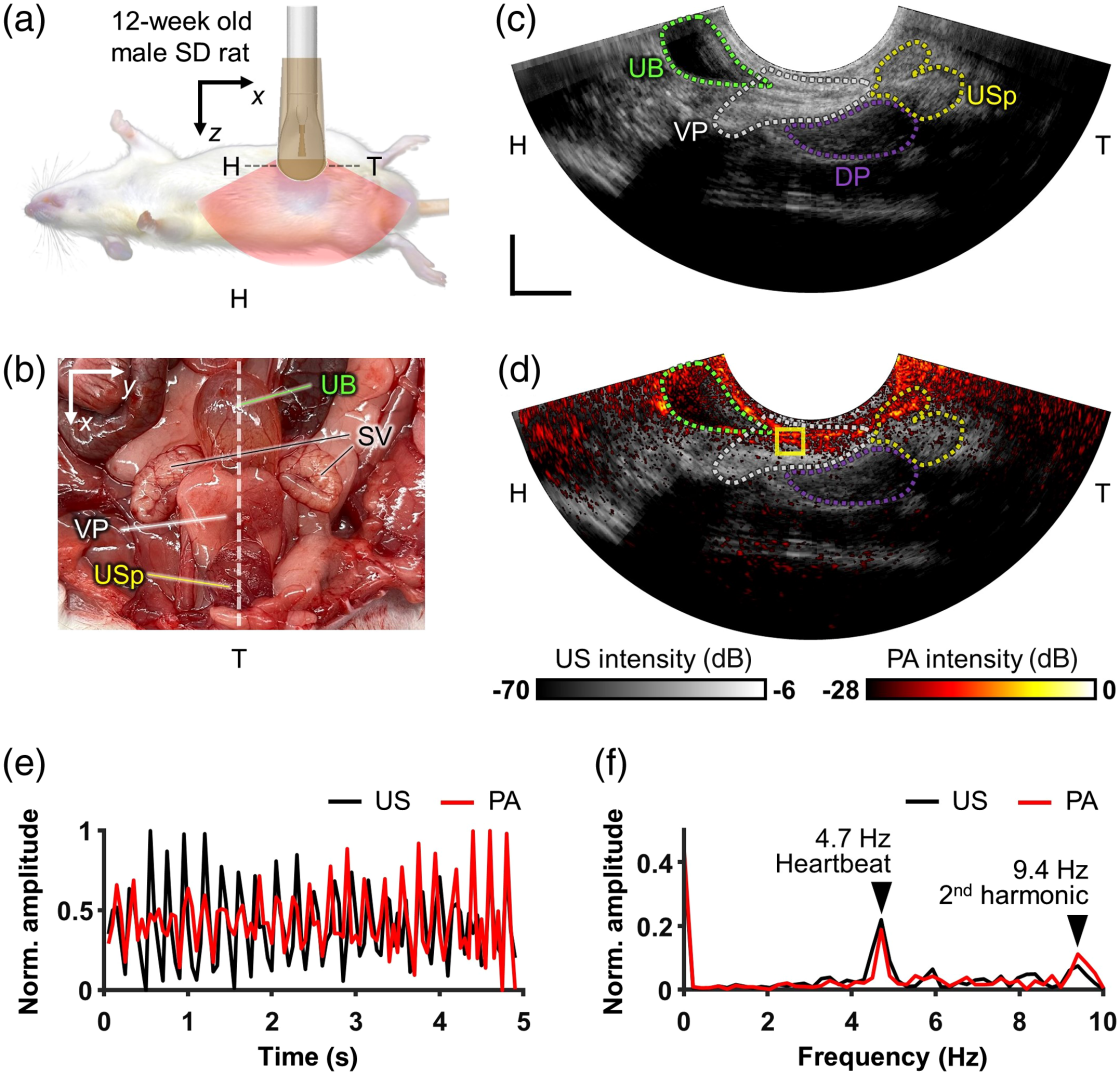
Arterial pulsation in a murine prostate monitored *in vivo* with EPAUSI: (a) illustration of a transabdominal imaging setting of a male rat in a supine position. (b) Anatomical photograph of a murine male reproductive organ. The dotted line indicates the imaging plane of the probe. (c) Harmonic US B-mode image in the sagittal plane. (d) Overlaid PA and harmonic US image corresponding to (c). (e) Time-resolved normalized US and PA amplitude change in prostatic junction (yellow box in panels (c) and (d); Video 2). (f) US and PA temporal frequency spectra corresponding to (e). UB, urinary bladder; SV, seminal vesicle; VP, ventral prostate; DP, dorsal prostate; and USp, urethral sphincter. Scale bar = 10 mm.

We evaluated the *in vivo* imaging capabilities of EPAUSI by imaging the hemodynamics in the male reproductive organ of a rat. All experimental procedures involving animals were conducted in compliance with the protocol approved by the Institutional Animal Care and Use Committee of Pohang University of Science and Technology (Protocol POSTECH-2021-0052-C2, approved on February 17, 2022). A healthy 12-week-old male Sprague Dawley rat with a body weight of 347 g was initially anesthetized in an induction chamber with 3% to 5% isoflurane/$O_{2}$ at a flow rate of 1.5  L/min, using a preclinical anesthetic gas system (VIP 3000 Veterinary Vaporizer, Midmark, Ohio, United States). The rat was supplied with a continuous stream of anesthetic gas through a customized nasal cap. About 75% of its abdominal hair was removed using clippers and depilatory cream, and the rat was placed on a heating pad in a supine position to expose the lower abdomen. An EPAUSI probe was placed over the lower abdomen of the rat after applying ultrasonic gel for PAUS imaging. The probe was aligned on the sagittal plane with the left side directed toward the cranial direction [refer to Fig. 7(a)]. Below the probe, the male murine genitalia were located, as evidenced by the post-experiment autopsy image of the organ’s arrangement [refer to Fig. 7(b)]. The imaging plane encompassed the urinary bladder (UB), ventral prostate (VP), and urethra, with each organ being distinctly visible in the harmonic US image, showing unique echogenicity and structure [refer to Fig. 7(c)].

Upon laser excitation, 700 nm PA and harmonic US images of the site were simultaneously displayed at a real-time frame rate of 20 Hz [refer to Fig. 7(d)]. In the center of both real-time views, we observed a repetitive pulsating motion at the boundary of the ventral and dorsal prostate junction at a depth of 8 mm (see Video 2). The prostate is a highly perfused organ with numerous interconnected blood vessels. Near its anatomical correspondence with the urethral axis, the identified vasculature appears to be a major branch from the central prostatic artery. Scattered PA contrast was also observed around the prostatic region, contributing to the subsidiary prostatic microvascular network. As we quantified the temporal amplitude changes in both PA and US images by comparing small regions of interest (ROI) at the pulsating site, we noticed synchronization between PA and US signals, evidenced by identical peaks [refer to Fig. 7(e)]. The signal changes were further analyzed by transforming them into temporal frequency spectra using fast Fourier transform (MATLAB R2021, Mathworks, Massachusetts, United States).^71^ These analyses revealed peaks at 4.7 and 9.4 Hz in both the US and PA spectra, representing the fundamental heartbeat frequency and its second harmonic [refer to Fig. 7(f)]. In summary, these findings confirm the practicality of our video-rate EPAUSI probe for capturing dynamic hemodynamics in live subjects.

### 3.5 Hemoglobin Oxygen Saturation (${\mathrm{sO}}_{2}$) Mapping of a Human Blood Vessel *In Vivo*

We further demonstrated the feasibility of EPAUSI by imaging representative blood vessels from various body parts of a healthy volunteer. These experiments were conducted in strict accordance with protocols approved by the Institutional Review Board of Pohang University of Science and Technology (Protocol POSTECH-PRIB-2023-R009, approved on April 3, 2023). Safety goggles were worn by both the examinee and the examiner to ensure laser safety, and the laser density was controlled to not exceed $25\;\mathrm{mJ}/{\mathrm{cm}}^{2}$ at 700 nm, complying with the ANSI MPE criterion. Various body parts of the examinee, including the neck, upper limb, and lower limb, were imaged by placing the EPAUSI probe in cutaneous contact with US gel. This allowed for the visualization of subcutaneous major vessels such as the carotid artery, jugular vein, radial artery, and popliteal vein. We conducted 20 Hz PAUS bimodal imaging, with PA images acquired at different laser wavelengths (690, 730, 756, and 796 nm) in series. Out of the acquired multispectral PA images, we decoupled oxyhemoglobin and deoxyhemoglobin via spectral unmixing, referring to the molecular extinction coefficient of hemoglobin (source: Oregon Medical Laser Center, Oregon, United States). We calculated pixelwise oxygen saturation (${\mathrm{sO}}_{2}$) by dividing oxyhemoglobin by the total hemoglobin, and the ${\mathrm{sO}}_{2}$ map was thresholded based on the spatially coherent 866 nm PA pixel intensity. This thresholded ${\mathrm{sO}}_{2}$ map was overlaid onto the ultrasound image, providing both structural and photochemical features of the vessels. To quantify sO2, we manually labeled ${\mathrm{sO}}_{2}$ pixels neighboring the vascular contrasts, and their upper 75% means were measured as nominal ${\mathrm{sO}}_{2}$ values. For each blood vessel, five independent measurements of ${\mathrm{sO}}_{2}$ were taken from consecutive ${\mathrm{sO}}_{2}$ frames, and the statistical presentation included calculating their mean values and standard errors.

**Fig. 8:**
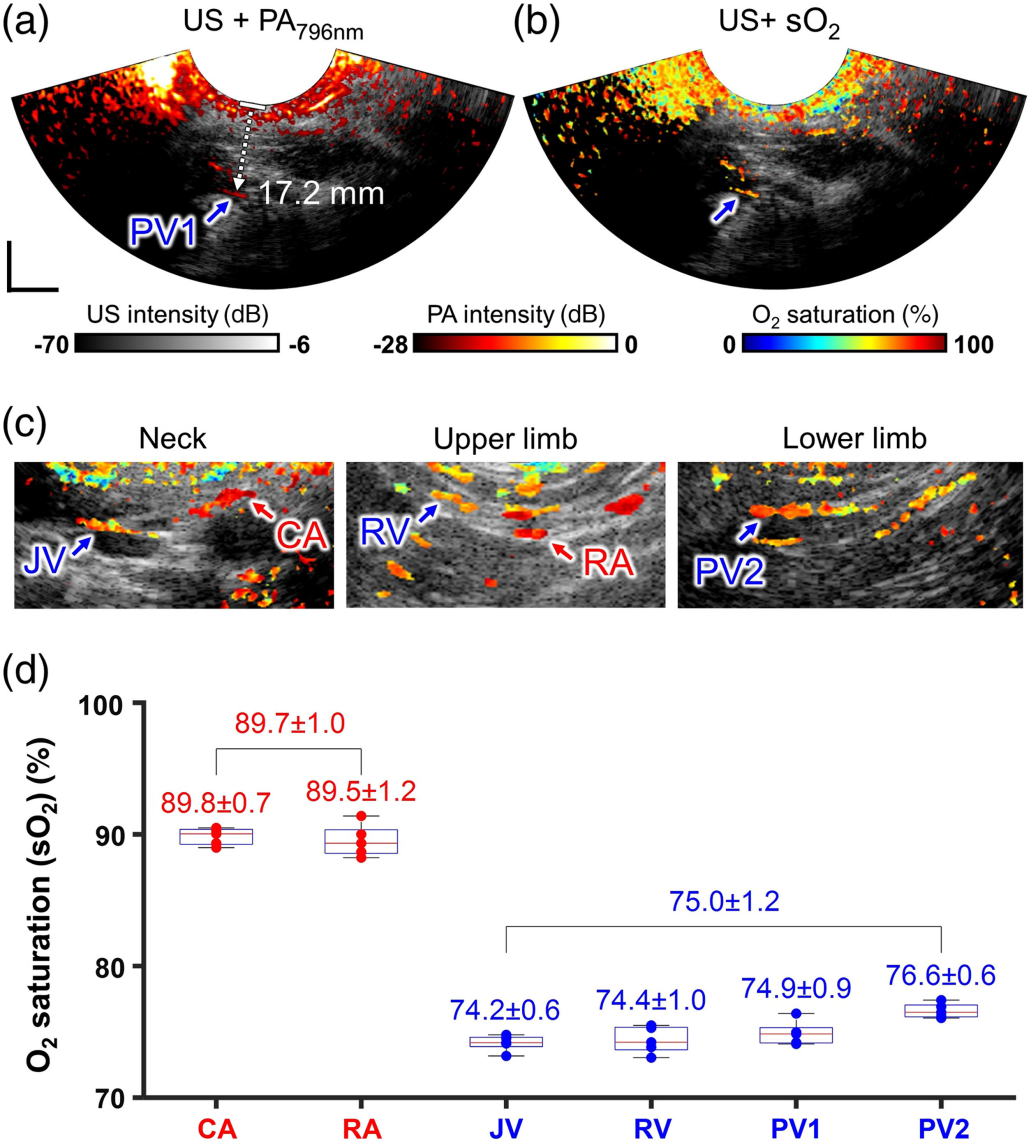
Human *in vivo* transcutaneous multispectral EPAUSI: (a) overlaid harmonic US B-mode and 796 nm PA images of a popliteal vein at a depth of 17.2 mm. (b) A corresponding oxygen saturation (sO2) map overlaid on the harmonic US B-mode image. (c) Series of sO2 maps identified from major vessels in neck, upper limb, and lower limb. (d) Quantitative statistics of nominal sO2 classified into arterial and venous group (two arteries, four veins, n=5). CA, carotid artery; RA, radial artery; JV, jugular vein; RV, radial vein; PV1, popliteal vein 1; and PV2, popliteal vein 2. Scale bar = 10 mm.

Among the numerous PA images, we identified the deepest vascular contrast at a depth of 17.2 mm, originating from the lower boundary of a popliteal vein [Fig. 8(a)]. Both the upper and lower boundaries were confirmed in the PA image, and their appearances were also presented in the subsequent ${\mathrm{sO}}_{2}$ images at the same depth [Fig. 8(b)]. The nominal ${\mathrm{sO}}_{2}$ was measured to be 76.7%±1.4%, a value well within the known range of oxygen saturation in venous flow. Similar strong PA signals and highly correlating ${\mathrm{sO}}_{2}$ values were observed for the rest of the vessels [Fig. 8(c)]. In the case of larger vessels, such as the jugular vein or carotid artery, PA contrasts were only visible at the upper boundary of the vessels, indicating both strong optical attenuation from the blood within the lumen and an enhanced limited view effect due to the large vessel diameter. Interestingly, it was possible to classify the imaged vessels as arteries or veins based solely on their nominal ${\mathrm{sO}}_{2}$ values [see Fig. 8(d)]. The ${\mathrm{sO}}_{2}$ levels were measured to be high at 89.9% and 88.7% for the carotid artery and radial artery, respectively. By contrast, the veins exhibited comparatively lower ${\mathrm{sO}}_{2}$ levels, with measurements of 74.9%, 75.1%, 76.7%, and 77.2% for the jugular vein, radial vein, and two popliteal veins, respectively. The high consistency in ${\mathrm{sO}}_{2}$ levels within the same vascular group, both across measurements and between different vessels, underscores the robustness of multispectral EPAUSI. This suggests its potential applicability in measuring ${\mathrm{sO}}_{2}$ in deep reproductive organs, which could efficiently capture hypoxic lesions commonly found in reproductive diseases, such as cancer or endometrioma.

## 4 Conclusion

In this study, we have developed a video-rate simultaneous endocavity PA and harmonic US imaging system designed for the diagnosis of gynecologic or urologic pathology via physically confined vaginal or rectal canals. This innovative system integrates a commercially available endocavity US probe with a miniaturized light delivery system consisting of a single large core fiber and a line beam engineered diffuser, resulting in a 25 mm thickness and ensuring high laser coupling efficiency. The system offers fine spatial resolution in both the radial (341  μm) and angular (1.23 deg) dimensions, combined with a temporal resolution of 20 Hz. These features prove highly advantageous for conducting multispectral PA imaging and capturing dynamic changes. The system’s capabilities were clearly demonstrated through various applications, including spectral unmixing of MB and ICG *in vitro*, ICG perfusion studies in an *ex vivo* porcine ovary, and monitoring of prostatic hemodynamics in an *in vivo* male rat. Furthermore, our functional EPAUSI successfully assessed the levels of ${\mathrm{sO}}_{2}$ in the arteries and veins of a healthy volunteer, showcasing its potential for identifying pathological hypoxia in reproductive organs. We believe that EPAUSI holds promise in enhancing diagnostic accuracy in cases of genital pathology, particularly in the diagnosis of conditions such as cancers or endometriosis, by revealing previously undetected signs of vascular changes.

## 5 Appendix: Video captions

The following videos are mentioned in the text:

Video 1 ICG perfusion dynamics in 20 Hz PA images (MP4, 6.95 MB [URL: https://doi.org/10.1117/1.JBO.29.S1.S11528.s1]).
Video 2 Prostatic pulsation recorded in 20 Hz PA and US images (MP4, 7.0 MB [URL: https://doi.org/10.1117/1.JBO.29.S1.S11528.s2]).

## Supplementary Material







## Data Availability

Data will be made available on request.

## References

[1] Sung H. et al., “Global cancer statistics 2020: GLOBOCAN estimates of incidence and mortality worldwide for 36 cancers in 185 countries,” Cancer J. Clin. 71(3), 209–249 (2021). 10.3322/caac.21660 33538338

[2] Dähnert W. et al., “Prostatic evaluation by transrectal sonography with histopathologic correlation: the echopenic appearance of early carcinoma,” Radiology 158(1), 97–102 (1986). RADLAX 0033-8419 10.1148/radiology.158.1.3510032 3510032

[3] Sassone A. M. et al., “Transvaginal sonographic characterization of ovarian disease: evaluation of a new scoring system to predict ovarian malignancy,” Obstet. Gynecol. 78(1), 70–76 (1991). 2047071

[4] Shinohara K. Wheeler T. M. Scardino P. T., “The appearance of prostate cancer on transrectal ultrasonography: correlation of imaging and pathological examinations,” J. Urol. 142(1), 76–82 (1989). 10.1016/S0022-5347(17)38666-4 2659828

[5] Modesitt S. C. et al., “Risk of malignancy in unilocular ovarian cystic tumors less than 10 centimeters in diameter,” Obstet. Gynecol. 102(3), 594–599 (2003). 10.1016/S0029-7844(03)00670-7 12962948

[6] Rodriguez L. V. Terris M. K., “Risks and complications of transrectal ultrasound guided prostate needle biopsy: a prospective study and review of the literature,” J. Urol. 160(6 Part 1), 2115–2120 (1998). 10.1016/S0022-5347(01)62255-9 9817335

[r7] Harvey C. et al., “Applications of transrectal ultrasound in prostate cancer,” Br. J. Radiol. 85(special_issue_1), S3–S17 (2012). BJRAAP 0007-1285 10.1259/bjr/56357549 22844031 PMC3746408

[8] Applewhite J. C. et al., “Transrectal ultrasound and biopsy in the early diagnosis of prostate cancer,” Cancer Control 8(2), 141–150 (2001). 10.1177/107327480100800204 11326168

[9] Terris M. K. et al., “Efficacy of transrectal ultrasound for identification of clinically undetected prostate cancer,” J. Urol. 146(1), 78–84 (1991). 10.1016/S0022-5347(17)37718-2 1711589

[10] Chen-Dixon K. et al., “Effectiveness of ultrasound for endometriosis diagnosis,” Curr. Opin. Obstet. Gynecol. 34(5), 324–331 (2022). COOGEA 1040-872X 10.1097/GCO.0000000000000812 36036477

[11] Park B. et al., “3D wide-field multispectral photoacoustic imaging of human melanomas in vivo: a pilot study,” J. Eur. Acad. Dermatol. Venereol. 35(3), 669–676 (2021). JEAVEQ 0926-9959 10.1111/jdv.16985 33037671

[r12] Kim J. et al., “Multiparametric photoacoustic analysis of human thyroid cancers in vivo,” Cancer Res. 81(18), 4849–4860 (2021). CNREA8 0008-5472 10.1158/0008-5472.CAN-20-3334 34185675

[r13] Karlas A. et al., “Optoacoustic imaging in endocrinology and metabolism,” Nat. Rev. Endocrinol. 17(6), 323–335 (2021). 10.1038/s41574-021-00482-5 33875856

[r14] Andreev V. G. et al., “Optoacoustic tomography of breast cancer with arc-array transducer,” Proc. SPIE 3916, 36–47 (2000). 10.1117/12.386339

[r15] Nyayapathi N. Xia J., “Photoacoustic imaging of breast cancer: a mini review of system design and image features,” J. Biomed. Opt. 24(12), 121911 (2019). JBOPFO 1083-3668 10.1117/1.JBO.24.12.121911 31677256 PMC7005545

[r16] Schoustra S. M. et al., “Twente Photoacoustic Mammoscope 2: system overview and three-dimensional vascular network images in healthy breasts,” J. Biomed. Opt. 24(12), 121909 (2019). JBOPFO 1083-3668 10.1117/1.JBO.24.12.121909 31650741 PMC7005569

[r17] Manohar S. Dantuma M., “Current and future trends in photoacoustic breast imaging,” Photoacoustics 16, 100134 (2019). 10.1016/j.pacs.2019.04.004 31871887 PMC6909206

[r18] Choi W. et al., “Three-dimensional multistructural quantitative photoacoustic and US imaging of human feet in vivo,” Radiology 303(2), 467–473 (2022). RADLAX 0033-8419 10.1148/radiol.211029 35191741

[r19] Fasoula N.-A. et al., “Non-invasive multispectral optoacoustic tomography resolves intrahepatic lipids in patients with hepatic steatosis,” Photoacoustics 29, 100454 (2023). 10.1016/j.pacs.2023.100454 36794122 PMC9922962

[20] Regensburger A. P. et al., “Detection of collagens by multispectral optoacoustic tomography as an imaging biomarker for Duchenne muscular dystrophy,” Nat. Med. 25(12), 1905–1915 (2019).1078-8956 10.1038/s41591-019-0669-y 31792454

[21] Choi W. et al., “Clinical photoacoustic imaging platforms,” Biomed. Eng. Lett. 8, 139–155 (2018). 10.1007/s13534-018-0062-7 30603199 PMC6208525

[r22] Attia A. B. E. et al., “A review of clinical photoacoustic imaging: current and future trends,” Photoacoustics 16, 100144 (2019). 10.1016/j.pacs.2019.100144 31871888 PMC6911900

[23] Park B. Kim C. Kim J., “Recent advances in ultrasound and photoacoustic analysis for thyroid cancer diagnosis,” Adv. Phys. Res. 2(4), 2200070 (2023). 10.1002/apxr.202200070

[24] Kim D. et al., “An ultraviolet-transparent ultrasound transducer enables high-resolution label-free photoacoustic histopathology,” Laser Photonics Rev. 18(2), 2300652 (2024). 10.1002/lpor.202300652

[r25] Park E. et al., “Azimuth mapping of fibrous tissue in linear dichroism-sensitive photoacoustic microscopy,” Photoacoustics 31, 100510 (2023). 10.1016/j.pacs.2023.100510 37228578 PMC10203768

[r26] Yao J. et al., “Breaking the speed limits in photoacoustic microscopy,” Photoacoustics 32, 100541 (2023). 10.1016/j.pacs.2023.100541 37692756 PMC10492007

[r27] Hwang S. H. et al., “3D printed multi-growth factor delivery patches fabricated using dual-crosslinked decellularized extracellular matrix-based hybrid inks to promote cerebral angiogenesis,” Acta Biomater. 157, 137–148 (2023). 10.1016/j.actbio.2022.11.050 36460287

[r28] Yoo J. et al., “Switchable preamplifier for dual modal photoacoustic and ultrasound imaging,” Biomed. Opt. Express 14(1), 89–105 (2023). BOEICL 2156-7085 10.1364/BOE.476453 36698663 PMC9842014

[29] Kim J. et al., “High-resolution photoacoustic/ultrasound imaging of the porcine stomach wall: an ex vivo feasibility study,” Biomed. Opt. Express 12(11), 6717–6729 (2021). BOEICL 2156-7085 10.1364/BOE.441241 34858676 PMC8606154

[30] Jeon S. et al., “Review on practical photoacoustic microscopy,” Photoacoustics 15, 100141 (2019). 10.1016/j.pacs.2019.100141 31463194 PMC6710377

[r31] Kim J. et al., “Deep learning acceleration of multiscale superresolution localization photoacoustic imaging,” Light: Sci. Appl. 11(1), 131 (2022). 10.1038/s41377-022-00820-w 35545614 PMC9095876

[r32] Park J. et al., “Quadruple ultrasound, photoacoustic, optical coherence, and fluorescence fusion imaging with a transparent ultrasound transducer,” Proc. Natl. Acad. Sci. 118(11), e1920879118 (2021). 10.1073/pnas.1920879118 33836558 PMC7980418

[r33] Baik J. W. et al., “Super wide-field photoacoustic microscopy of animals and humans in vivo,” IEEE Trans. Med. Imaging 39(4), 975–984 (2019). ITMID4 0278-0062 10.1109/TMI.2019.2938518 31484110

[r34] Kwon N. et al., “Hexa-BODIPY-cyclotriphosphazene based nanoparticle for NIR fluorescence/photoacoustic dual-modal imaging and photothermal cancer therapy,” Biosens. Bioelectron. 216, 114612 (2022). BBIOE4 0956-5663 10.1016/j.bios.2022.114612 35952434

[r35] Ding Y. et al., “Surfactant-stripped semiconducting polymer micelles for tumor theranostics and deep tissue imaging in the NIR-II window,” Small 18(6), 2104132 (2022). SMALBC 1613-6810 10.1002/smll.202104132 34850550

[r36] Maji D. et al., “Copper-catalyzed covalent dimerization of near-infrared fluorescent cyanine dyes: synergistic enhancement of photoacoustic signals for molecular imaging of tumors,” Anal. Sens. 2(1), e202100045 (2022). 10.1002/anse.202100045 37621644 PMC10448761

[37] Park J. et al., “Bi-modal near-infrared fluorescence and ultrasound imaging via a transparent ultrasound transducer for sentinel lymph node localization,” Opt. Lett. 47(2), 393–396 (2022). OPLEDP 0146-9592 10.1364/OL.446041 35030614

[38] Manohar S. Razansky D., “Photoacoustics: a historical review,” Adv. Opt. Photonics 8(4), 586–617 (2016). AOPAC7 1943-8206 10.1364/AOP.8.000586

[r39] Wang L. V., “Prospects of photoacoustic tomography,” Med. Phys. 35(12), 5758–5767 (2008). MPHYA6 0094-2405 10.1118/1.3013698 19175133 PMC2647010

[r40] Wang L. V. Yao J., “A practical guide to photoacoustic tomography in the life sciences,” Nat. Methods 13(8), 627–638 (2016).1548-7091 10.1038/nmeth.3925 27467726 PMC4980387

[r41] Oh D. et al., “Contrast agent-free 3D renal ultrafast doppler imaging reveals vascular dysfunction in acute and diabetic kidney diseases,” Adv. Sci. 10(36), 2303966 (2023). 10.1002/advs.202303966 PMC10754092 37847902

[42] Kim W. et al., “Wide-field three-dimensional photoacoustic/ultrasound scanner using a two-dimensional matrix transducer array,” Opt. Lett. 48(2), 343–346 (2023). OPLEDP 0146-9592 10.1364/OL.475725 36638453

[43] Ahn J. et al., “In vivo photoacoustic monitoring of vasoconstriction induced by acute hyperglycemia,” Photoacoustics 30, 100485 (2023). 10.1016/j.pacs.2023.100485 37082618 PMC10112177

[r44] Park B. et al., “Functional photoacoustic imaging: from nano-and micro-to macro-scale,” Nano Converg. 10(1), 29 (2023). 10.1186/s40580-023-00377-3 37335405 PMC10279631

[r45] Choi S. et al., “Deep learning enhances multiparametric dynamic volumetric photoacoustic computed tomography in vivo (DL-PACT),” Adv. Sci. 10(1), 2202089 (2023). 10.1002/advs.202202089 PMC9811490 36354200

[46] Ahn J. et al., “Fully integrated photoacoustic microscopy and photoplethysmography of human in vivo,” Photoacoustics 27, 100374 (2022). 10.1016/j.pacs.2022.100374 35646590 PMC9133750

[47] Choi W. et al., “Recent advances in contrast-enhanced photoacoustic imaging: overcoming the physical and practical challenges,” Chem. Rev. 123(11), 7379–7419 (2023). CHREAY 0009-2665 10.1021/acs.chemrev.2c00627 36642892

[r48] Park B. et al., “Shear-force photoacoustic microscopy: toward super-resolution near-field imaging,” Laser Photonics Rev. 16(12), 2200296 (2022). 10.1002/lpor.202200296

[49] Park B. et al., “Listening to drug delivery and responses via photoacoustic imaging,” Adv. Drug Deliv. Rev. 184, 114235 (2022). ADDREP 0169-409X 10.1016/j.addr.2022.114235 35346776

[50] Choi W. Oh D. Kim C., “Practical photoacoustic tomography: realistic limitations and technical solutions,” J. Appl. Phys. 127(23), 230903 (2020). JAPIAU 0021-8979 10.1063/5.0008401

[r51] Cho S. et al., “3D PHOVIS: 3D photoacoustic visualization studio,” Photoacoustics 18, 100168 (2020). 10.1016/j.pacs.2020.100168 32211292 PMC7082691

[52] MacCuaig W. M. et al., “Development of multispectral optoacoustic tomography as a clinically translatable modality for cancer imaging,” Radiol. Imaging Cancer 2(6), e200066 (2020). 10.1148/rycan.2020200066 33330850 PMC7706874

[r53] Taruttis A. Ntziachristos V., “Advances in real-time multispectral optoacoustic imaging and its applications,” Nat. Photonics 9(4), 219–227 (2015). NPAHBY 1749-4885 10.1038/nphoton.2015.29

[r54] Ntziachristos V. Razansky D., “Molecular imaging by means of multispectral optoacoustic tomography (MSOT),” Chem. Rev. 110(5), 2783–2794 (2010). CHREAY 0009-2665 10.1021/cr9002566 20387910

[r55] Gujrati V. Mishra A. Ntziachristos V., “Molecular imaging probes for multi-spectral optoacoustic tomography,” Chem. Commun. 53(34), 4653–4672 (2017). 10.1039/C6CC09421J 28387781

[r56] Yoon C. et al., “Motion compensation for 3D multispectral handheld photoacoustic imaging,” Biosensors 12(12), 1092 (2022). BISSED 0265-928X 10.3390/bios12121092 36551059 PMC9775698

[57] Yang J. Choi S. Kim C., “Practical review on photoacoustic computed tomography using curved ultrasound array transducer,” Biomed. Eng. Lett. 12, 19–35 (2022). 10.1007/s13534-021-00214-8 35186358 PMC8825902

[58] Lee C. et al., “Panoramic volumetric clinical handheld photoacoustic and ultrasound imaging,” Photoacoustics 31, 100512 (2023). 10.1016/j.pacs.2023.100512 37252650 PMC10208888

[59] Lee C. et al., “Three-dimensional clinical handheld photoacoustic/ultrasound scanner,” Photoacoustics 18, 100173 (2020). 10.1016/j.pacs.2020.100173 32215250 PMC7090348

[60] Horiguchi A. et al., “A pilot study of photoacoustic imaging system for improved real-time visualization of neurovascular bundle during radical prostatectomy,” Prostate 76(3), 307–315 (2016). 10.1002/pros.23122 26493623

[r61] Horiguchi A. et al., “Pilot study of prostate cancer angiogenesis imaging using a photoacoustic imaging system,” Urology 108, 212–219 (2017). 10.1016/j.urology.2017.07.008 28735020

[r62] Kothapalli S.-R. et al., “Simultaneous transrectal ultrasound and photoacoustic human prostate imaging,” Sci. Transl. Med. 11(507), eaav2169 (2019). STMCBQ 1946-6234 10.1126/scitranslmed.aav2169 31462508

[r63] Liu C. et al., “In vivo transrectal imaging of canine prostate with a sensitive and compact handheld transrectal array photoacoustic probe for early diagnosis of prostate cancer,” Biomed. Opt. Express 10(4), 1707–1717 (2019). BOEICL 2156-7085 10.1364/BOE.10.001707 31086699 PMC6484995

[64] Jang J. et al., “Transrectal ultrasound and photoacoustic imaging probe for diagnosis of prostate cancer,” Sensors 21(4), 1217 (2021). SNSRES 0746-9462 10.3390/s21041217 33572287 PMC7915711

[65] Salehi H. S. et al., “Design of optimal light delivery system for co-registered transvaginal ultrasound and photoacoustic imaging of ovarian tissue,” Photoacoustics 3(3), 114–122 (2015). 10.1016/j.pacs.2015.08.003 26640774 PMC4595518

[r66] Cong B. et al., “A low cost sensitive transrectal photoacoustic probe with single-fiber bright-field illumination for in vivo canine prostate imaging and real-time biopsy needle guidance,” IEEE Sens. J. 20(18), 10974–10980 (2020). ISJEAZ 1530-437X 10.1109/JSEN.2020.2993884

[r67] Yan Y. et al., “Endocavity ultrasound and photoacoustic system for fetal and maternal imaging: design, implementation, and ex-vivo validation,” J. Med. Imaging 8(6), 066001 (2021). JMEIET 0920-5497 10.1117/1.JMI.8.6.066001 PMC8577694 34778491

[68] Yang G. et al., “Optimizing light delivery through ball-shaped multimode fiber tips in co-registered photoacoustic and ultrasound endo-cavity imaging: simulation and experimental validation,” Photons Plus Ultrasound Imaging Sens. 2019, 465–471 (2019). 10.1117/12.2510914

[69] Prahl S., “Assorted spectra (Hemoglobin, Indocyanine Green, Methylene Blue),” https://omlc.org/spectra (1999).

[70] Akarçay H. G. et al., “Determining the optical properties of a gelatin-${\mathrm{TiO}}_{2}$ phantom at 780 nm,” Biomed. Opt. Express 3(3), 418–434 (2012). BOEICL 2156-7085 10.1364/BOE.3.000418 22435091 PMC3296531

[71] Li L. et al., “Single-impulse panoramic photoacoustic computed tomography of small-animal whole-body dynamics at high spatiotemporal resolution,” Nat. Biomed. Eng. 1(5), 0071 (2017). 10.1038/s41551-017-0071 29333331 PMC5766044

