# Supplementary material for: Video-rate endocavity photoacoustic/harmonic ultrasound imaging with miniaturized light delivery
Source: J Biomed Opt. 2024 Mar 19;29(Suppl 1):S11528. doi: 10.1117/1.JBO.29.S1.S11528 (PMC10949014; doi:10.1117/1.JBO.29.S1.S11528)
Supplement: Supplementary file 1 [file JBO_029_S11528_SD001.pdf]

Manuscript title: Video-Rate Endocavity Photoacoustic/Harmonic Ultrasound Imaging with Miniaturized Light Delivery

Fig. S1 Pulse-inversion harmonic US imaging for enhanced tissue contrast.

Fig. S2 PA and harmonic US elevational beam profile.

Table S1 EPAUSI hardware specification in detail

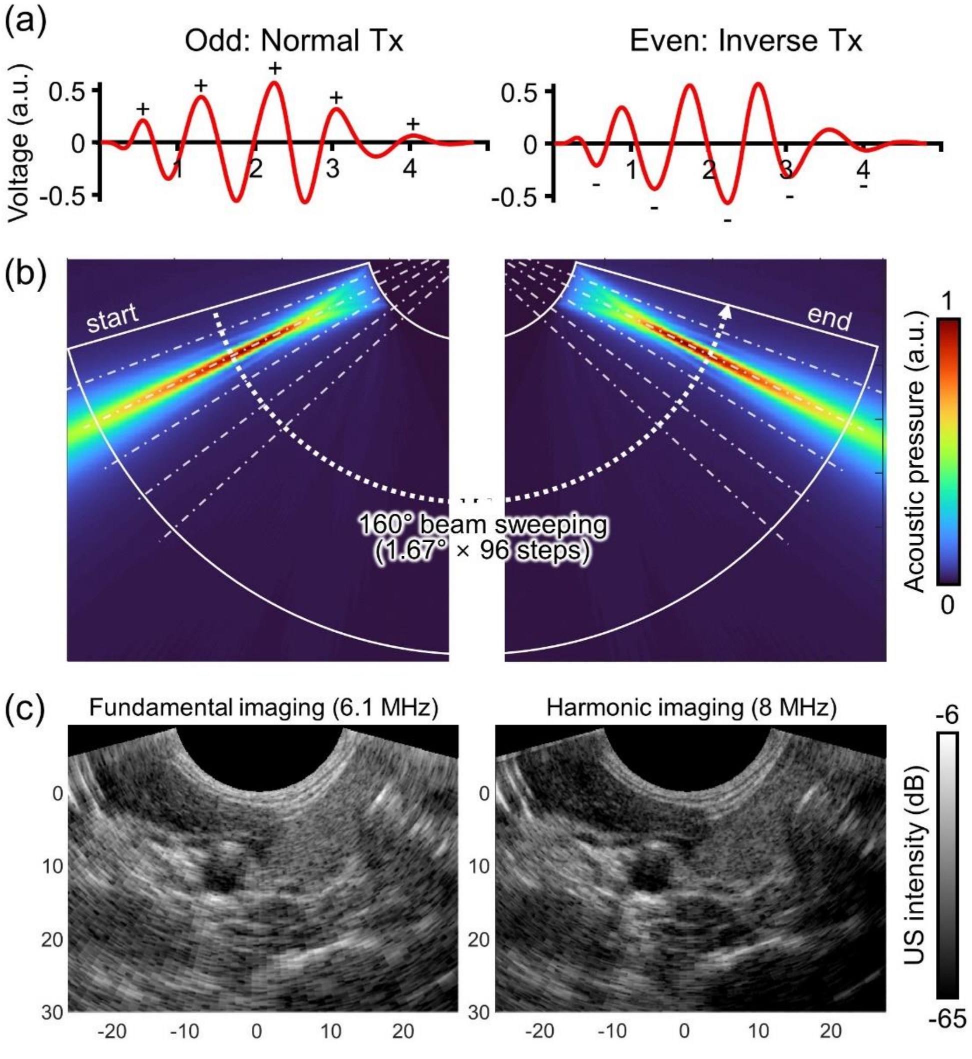

Fig S1. Pulse-inversion harmonic US imaging for enhanced tissue contrast. (a) Transmit wave profile of a designed normal and inverted pair (4 MHz, 2 half cycle). (b) A numerically simulated acoustic pressure density map for line-scan. A pair of normal and inverted beam is transmitted on identical scanline, and the beam is swept in 160° span within 96 steps. (c) Qualitative comparison between 6.1 MHz fundamental imaging and pulse-inversion harmonic imaging (8 MHz) of a human neck. The tissue boundary between neck muscle (left), carotid arterial wall (middle) and thyroid (right) is clearer in harmonic images.

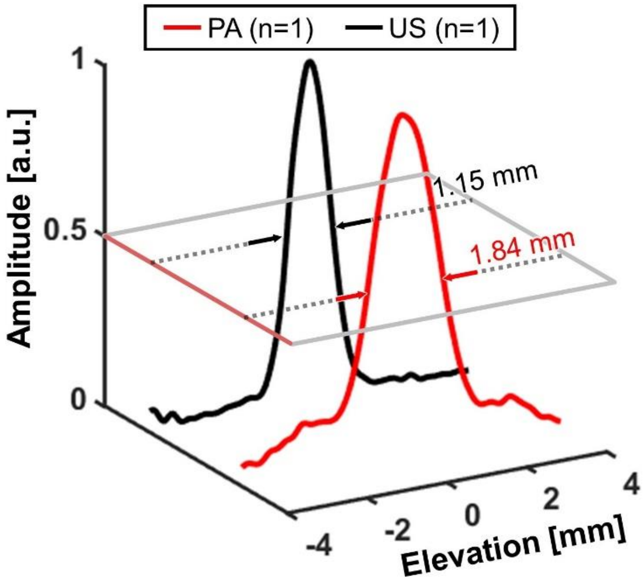

Fig. S2 PA and harmonic US elevational beam profile.

Table S1 EPAUSI hardware specification in detail

**Probe**	**6EIX, Humanscan, Republic of Korea**	
Center frequency, -6 dB bandwidth	6.11 MHz, 75%	
Number of elements	192	
Pitch	0.140 mm	
Field of view	161.6°	
Radius of curvature	10 mm	
Elevational length	6 mm	
Elevational focus	30 mm	
**Ultrasound system**	**Vantage 256-high frequency configuration, Verasonics, WA, USA**	
Active Tx/Rcv channel	256	
Multiplexer ratio	1:1 (100%)	
TX frequency (PA/US)	-	4 MHz
Sampling frequency (PA/US)	25 MHz	31.25 MHz
Transmit voltage (PA/US)	-	30 V
Number of Tx/Rcv events (per image, PA/US)	1	192
**Laser system**	**Photosonus M, Ekspla, Lithuania**	
PRF	20 Hz	
Pulse width	3-5 ns	
Wavelength range	660-1064 nm (signal)	
Output pulse energy	< 160 mJ	
